# English Hotels and Their Critics

**Published:** 1889-07-20

**Authors:** 


### The Hospital

A Weekly Institutional Journal of

Science, Medicine, Nursing, and Philanthropy.

*For Hospitals, Asylums, Medical Practitioners, Students, Nurses, Families, Parishes, Congregations, and the Charitable Public.*

Vol. VI. No. 147.] [July 20, 1889.

### English Hotels and Their Critics.

Under this heading we publish elsewhere a letter signed "Experientia Docet." The writer makes charges against English hotel-keepers which, if they are in any sense largely true, convict the accused, as a class, of disgraceful offences against common honesty, common-sense, and the health and happiness of their clients. He appeals through our columns to the medical profession, insisting that they ought to be well informed on the subject, and to warn their patients against the dangers and discomforts that await them when they submit themselves to the tender mercies of devouring caterers. He pictures, with sympathetic fervour, the case of a delicately-nurtured and carefully-dieted young lady who, after a tremendous journey of perhaps fifty or sixty miles in a well-padded first-class carriage, arrives at the station for which she is destined, longing for that universal solace of afflicted lady-kind—a cup of tea. Something which is called "tea" is presently forthcoming, but it is a "brew" from leaves which only cost one and twopence a pound, says our indignant correspondent; and even a coolie would blush through his dark skin if he were asked to offer such a decoction to the "refined" young woman who has just come by the train. The "thin" toast so dear to delicate appetites is not to be procured; but instead thereof a "flabby leaf of dough" is placed on the table, or, more disgusting still, a "flinty charcoal slab." We cannot but admire the discriminating choice of epithets displayed by our correspondent, which, in however slight a degree they may be the equivalents of the actual facts, are undoubtedly very striking and effective in themselves. The "flabby leaf of dough," as descriptive of the finer kind of hotel toast, and the "flinty slab of charcoal," it would be difficult to surpass. We commend them to the consideration of Mr. Andrew Lang.

The *table d'hote* to which hungry creatures of the male sex look forward with so much gusto, and to which, also, it must be confessed, the average British woman usually contrives to do ample justice, fills our correspondent with undisguised and unmitigated horror. The soup, the rich and savoury oxtail, he declares to be a dreadful infusion of venerable caudal appendages on which bluebottles have for a long time daily held high revel, until the evidences of immediate decomposition compelled the butcher to sell them to his friend the hotel-keeper at a "great reduction." The fish, like all the other eatables, is contracted for, and may be prime and fresh or "offal" several days old, the latter being considered peculiarly suitable for commercial men and yellow-plushes. The "rissoles" are as artless and mysterious as some of the fair ones who partake of them. Even the sirloin, that dish of aldermen and kings, is no longer the glorious "roast beef of old England," but the lean, scraggy and wretched "old beef" roasted, alas, in England, and intended for Englishmen to eat. The miserable butcher who thus sullies the fair fame of his country has shares in the new hotel, and so gets double dividends by selling to the proprietor joints which nobody else would buy. Finally, the coffee and the wines are said to be "vile medicaments," and "liquors supplied by syndicates," whose only object appears to be to sell at a high price "vintages" which, if health considerations prevailed, nobody would ever drink at all. The writer contends that "modern hotel catering is a gigantic fraud," that the people who visit hotels are "cheated of their daily bread," and ruined in digestion and health by the unscrupulous greed of those who prey on their defenceless condition.

All this sounds very dreadful. But is it true? Is it true universally, or is it partly true, or is it not true at all? We give our correspondent's letter publicity because of the wide-reaching extent and national importance of the subject. But for our part we confess that we think his "charges and allegations," inso far as they apply to the hotel-keepers of England as a class, are as destitute of foundation as they can well be. Nothing is easier than to make sweeping accusations against whole classes of people who are practically without means of defence. A man should, however, think twice before he strives to convict many thousands of his fellow-countrymen of offences which, if proved, would well merit the punishment of the felon's cell. Few things are more cowardly than to stand behind a safe wall and to fire at a whole company of unarmed persons through a porthole. This, it seems to us, is what our correspondent has done. It may be, and probably is the fact, that at one or two hotels his experience has been unfortunate. But because there was one Palmer in England, would it be just to call our countrymen a race of poisoners? Or, because one soldier has been known to run away, would it be true to say we are a nation of cowards?

The Press, we conceive, has high public duties to perform. One of those duties undoubtedly is to give the freest possible publicity to all kinds of evils and abuses which are of sufficient importance to be worthy of public attention. The hotels of this country are not only places of sojourn for men whose business carries them from town to town all the year round; but they are used as health and pleasure resorts by many thousands to whom health is of prime importance, and whose pleasures are so few that they ought to be of the most perfect possible kind. If, therefore, there were any justification for "Experientia's" comprehensive and grave charges, we should feel it our duty, alike in the interests of public morals and of individual health and happiness, to do our utmost to probe the matter to the bottom. But our experience lies all the other way. The present writer has been familiar with English and Scotch hotels for the last twenty years. He has also some personal knowledge of continental hotels. Speaking generally, it may be affirmed that the English hotels compare in most respects very favourably indeed with their continental rivals. No doubt many persons have a decided preference for the best French cookery; and for that there is much to be said. Equally, no doubt, the average continental hotel is a more economical place than its fellow of this country. But, on the whole, an Englishman will prefer a month or six weeks at a good English hotel to the same period spent at one place on the Continent. The writer expresses the decided conviction, based upon a fairly long and extensive experience, that the proprietors and managers of the best class of hotels in this country are competent, honest, and obliging people, and that at least 80 per cent. of British hotels are conducted in the highest style of excellence.

It is, of course, quite impossible that the experience of one or two persons, either for or against any business which is as wide as the nation, can be conclusive. A much more extensive experience must be included before any authoritative utterance can be made. At the present time most country and seaside hotels are full, and are likely to remain so for the next three months. Visitors, therefore, will have ample opportunities of judging for themselves how far they are catered for with honesty, skill, and success. We are all for truth and fair play; and if there are any, like our correspondent, "Experientia," who think they have just ground of complaint, we shall be glad to give them as much space as our columns will reasonably allow. If, on the other hand, there are persons who prove by different experience that a good English hotel is a thoroughly healthy and comfortable place, though rather expensive withal, we shall give them also the temporary hospitality of our pages on the same conditions of reasonable limitation. Two chief characteristics of Englishmen are straightforwardness and love of fair-play. We have given our correspondent's views fully, and we have expressed our judgment thereupon. If other combatants feel that they would like to take part in the fray, the stage is now clear.

